# The HRDC domain of *E. coli* RecQ helicase controls single-stranded DNA translocation and double-stranded DNA unwinding rates without affecting mechanoenzymatic coupling

**DOI:** 10.1038/srep11091

**Published:** 2015-06-11

**Authors:** Gábor M. Harami, Nikolett T. Nagy, Máté Martina, Keir C. Neuman, Mihály Kovács

**Affiliations:** 1 Department of Biochemistry, ELTE-MTA “Momentum” Motor Enzymology Research Group, Eötvös Loránd University, Pázmány P. s. 1/c, H-1117 Budapest, Hungary; 2 Laboratory of Molecular Biophysics, National Heart, Lung and Blood Institute, National Institutes of; 3 Health, Bethesda, Maryland 20892, USA

## Abstract

DNA-restructuring activities of RecQ-family helicases play key roles in genome maintenance. These activities, driven by two tandem RecA-like core domains, are thought to be controlled by accessory DNA-binding elements including the helicase-and-RnaseD-C-terminal (HRDC) domain. The HRDC domain of human Bloom’s syndrome (BLM) helicase was shown to interact with the RecA core, raising the possibility that it may affect the coupling between ATP hydrolysis, translocation along single-stranded (ss)DNA and/or unwinding of double-stranded (ds)DNA. Here, we determined how these activities are affected by the abolition of the ssDNA interaction of the HRDC domain or the deletion of the entire domain in *E. coli* RecQ helicase. Our data show that the HRDC domain suppresses the rate of DNA-activated ATPase activity in parallel with those of ssDNA translocation and dsDNA unwinding, regardless of the ssDNA binding capability of this domain. The HRDC domain does not affect either the processivity of ssDNA translocation or the tight coupling between the ATPase, translocation, and unwinding activities. Thus, the mechanochemical coupling of *E. coli* RecQ appears to be independent of HRDC-ssDNA and HRDC-RecA core interactions, which may play roles in more specialized functions of the enzyme.

RecQ-family helicases, which are key players in genome maintenance in both bacteria and eukaryotes, perform ssDNA translocation and dsDNA unwinding driven by ATP hydrolysis^1^,^2^,^3^. What makes RecQ enzymes different from other helicases is their unique capability to unwind various non-canonical DNA structures that arise during DNA replication, recombination and repair^1^,^2^,^3^. *E. coli* (*Ec*) RecQ helicase, the prototypic family member, is required for the processing of late replication intermediates and stalled replication forks^4^,^5^,^6^, exerts both pro- and anti-recombination activities^7^,^8^ and suppresses illegitimate recombination^9^. Mutations in three of five human RecQ homologs (BLM, WRN (Werner syndrome helicase) and RecQ4) cause severe syndromes bearing the common feature of loss of genomic integrity, highlighting the essential role of RecQ helicases in genome maintenance^1^,^3^.

The capability of RecQ helicases to process complex DNA structures is associated with a conserved domain architecture shared by *Ec* RecQ, human BLM and WRN enzymes, as well as at least one RecQ homolog in almost all investigated organisms (Fig. 1)^10^. Two conserved RecA-like domains harbor the ATPase cleft and drive 3′–5′-directed ssDNA translocation activity^2^,^11^. In most RecQ helicases, these domains are followed by a zinc-binding domain, which appears essential for the structural stability of the enzymes and contributes to DNA binding^12^,^13^,^14^. Furthermore, many RecQ helicases contain the auxiliary winged-helix (WHD) and HRDC domains^2^,^11^. The WHD mediates protein-DNA and protein-protein interactions and has been shown to facilitate DNA unwinding and contribute to substrate recognition^10^.

The HRDC domain, which is connected to the WHD via a flexible linker, also contributes to the substrate specificity and DNA binding affinity of RecQ helicases^2^,^11^,^15^,^16^,^17^. Despite their similar overall fold, HRDC domains of RecQ helicases show highly variable surface properties and ssDNA binding affinities^16^,^18^,^19^,^20^,^21^. For *Ec* RecQ, atomic structures have been solved separately for the HRDC-deleted helicase core and for the isolated HRDC domain (Fig. 1b)^16^,^22^. Atomic structures containing the RecQ helicase core together with the HRDC domain have been solved only for human BLM^23^ (PDB codes 4O3M, 4CDG, 4CGZ). In these structures the HRDC domain folds back onto the core and interacts with both RecA domains (Fig. 1c). Deletion of the BLM HRDC domain increased the ATPase activity of the enzyme while decreasing the rate of dsDNA unwinding^23^. These findings led to the proposition that the HRDC-core interaction is required for efficient coupling of the ATPase activity to dsDNA unwinding^23^.

Previous studies by us and others showed that RecQ and BLM helicases translocate on ssDNA with moderate processivity^24^,^25^,^26^,^27^. Translocation is tightly coupled to ATP hydrolysis, and the enzymes translocate 1- 2 nucleotide (nt) upon hydrolysis of one ATP molecule^24^,^25^,^26^. In addition we found that, similar to the situation in BLM, the *Ec* RecQ HRDC domain suppresses the ssDNA-activated ATPase activity of the enzyme^17^. These findings suggest a general role for HRDC domains in supporting coupling between ATPase and translocation/unwinding activities through interdomain interactions. To test this hypothesis, here we performed transient and steady-state kinetic measurements using wild-type (WT), HRDC point mutant and an HRDC deletion mutant *Ec* RecQ constructs to dissect the mechanism of ATPase-coupled translocation and unwinding and its modulation by HRDC domain interactions. We found that the HRDC domain slows down ssDNA translocation and dsDNA unwinding by suppressing the ATPase activity, but it does not influence the processivity of translocation and the coupling of the ATPase activity to translocation and unwinding. These results suggest that the interaction between the motor core and the HRDC domain is conserved among RecQ helicases and this interaction moderates the rate of ATPase-driven activities, but its effect on mechanochemical coupling may vary among RecQ homologs, according to the differing physiological roles of the enzymes.

## Results

### Protein constructs

**Figure 1:**
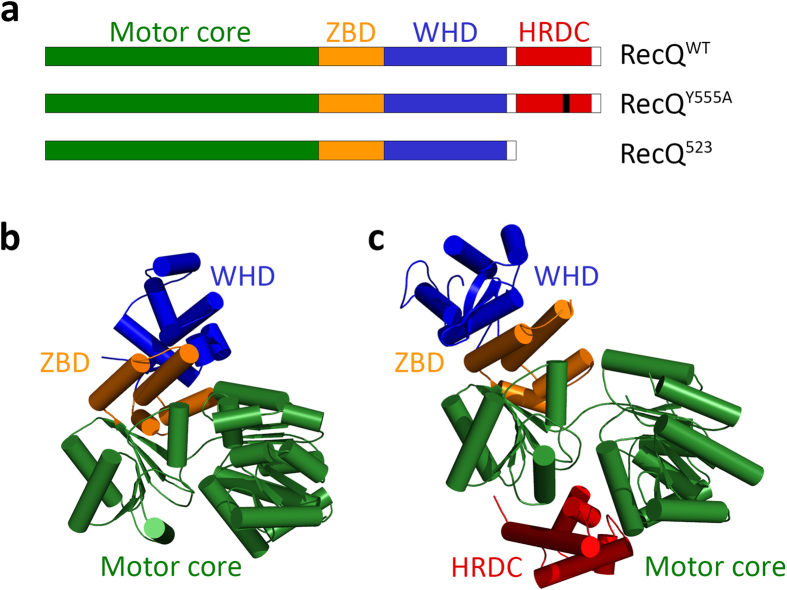
RecQ constructs used in this study (**a**) Schematic domain map of *Ec* RecQ constructs used in this study. The motor core comprises two RecA-like domains. ZBD, zinc-binding domain; WHD, winged-helix domain; HRDC, helicase-and-RNase-D-C-terminal domain. RecQ^Y555A^ harbors the Y555A point mutation in the HRDC domain (black bar). RecQ^523^ lacks the entire HRDC domain. (**b–c**) Crystal structures of a HRDC-deleted *Ec* RecQ construct (**b,** PDB code 1OYW) and a HRDC-containing human BLM construct (**c,** PDB code 4O3 M). Domains are colored as in **a**.

To dissect the role of the HRDC domain of *Ec* RecQ helicase in the enzymatic mechanism, we used three protein constructs: wild-type protein (RecQ^WT^), a construct in which the previously characterized Y555A point mutation abolishes the ssDNA-binding ability of the HRDC domain (RecQ^Y555A^)^16^, and a construct lacking the entire HRDC domain (RecQ^523^, comprising amino acids (a.a.) 1-523) (Fig. 1a). Circular dichroism measurements indicated proper folding of all constructs (Supplementary Fig. S1).

### ATPase suppression by the HRDC domain does not greatly affect ssDNA translocation processivity or ATPase-translocation coupling

**Figure 2:**
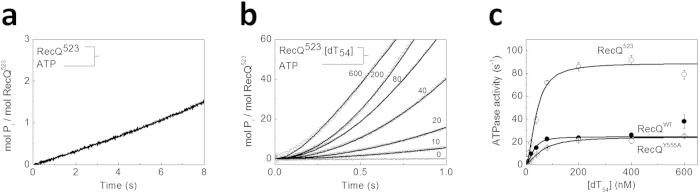
The HRDC domain suppresses RecQ ATPase activity (**a**) Kinetics of P_i_ generation from ATP (1 mM) by RecQ^523^ (20 nM) in the absence of DNA, followed by MDCC-PBP (5 μM) fluorescence in the stopped-flow. (**b**) Kinetic traces of P_i_ release by RecQ^523^ (as in panel **a** but in the presence of various dT_54_ concentrations, indicated in nM). Traces were analyzed using Supplementary equation (S1) to determine the steady-state ATPase activity values shown in (**c**) for RecQ^WT^ (solid circles), RecQ^Y555A^ (open diamonds) and RecQ^523^ (open circles). Solid lines in (**c**) show fits using the Hill equation. Determined parameters are listed in Table 1.

To monitor the ATPase activity of helicase constructs, we followed the kinetics of inorganic phosphate (P_i_) generation from ATP in a stopped-flow instrument. The amount of P_i_ produced was quantified using a fluorescently labeled phosphate binding protein (MDCC-PBP) (Supplementary Fig. S2)^24^,^25^,^26^,^28^,^29^. In the absence of DNA, traces of P_i_ generation were linear for all constructs in the presence of saturating ATP concentration (shown for RecQ^523^ in Fig. 2a). Steady-state DNA-free ATPase slopes for RecQ^WT^ were consistent with previous results determined by other methods^25^,^30^. The DNA-free ATPase activity (*k*_basal_) was not influenced by either the Y555A mutation or HRDC deletion (Table 1).

As shown previously, ssDNA markedly activates RecQ ATPase activity^30^,^31^. To assess the role of the HRDC domain in ssDNA-induced ATPase activation, we rapidly mixed ATP with RecQ constructs pre-mixed with increasing amounts of dT_54_, and monitored P_i_ release kinetics. Kinetic traces showed a lag followed by quasi-linear P_i_ generation for all constructs (shown for RecQ^523^ in Fig. 2b). The onset of the linear steady-state became faster with increasing DNA concentration with no sign of saturation, suggesting that the lag originated from an enzyme-DNA binding process induced by mixing with ATP (Fig. 2b). Our earlier study showed that ATP binds very rapidly to the enzyme^30^, ruling out that the lag in Fig. 2b originates from ATP binding. However, we recently showed that the presence of ATP, or its analogs, markedly increases the ssDNA affinity of RecQ^17^. Therefore, the lag can be explained by an ATP-induced increase in the fraction of ssDNA-bound RecQ molecules occurring upon stopped-flow mixing. This leads to a concomitant increase in the rate of P_i_ generation until reaching the steady-state.

We determined the apparent rate constant of the ATP-induced DNA binding process (*k*_b_) and the steady-state ATPase rate (*k*_ss_) at each dT_54_ concentration from the P_i_ generation transients (Fig. 2b) using the model described in Supplementary equation (S1). Fits to the dT_54_ concentration dependence of *k*_ss_ using the Hill equation revealed ssDNA-activated *k*_cat_ values (i.e. maximal *k*_ss_ at saturating dT_54_ concentration) in line with those determined previously using an NADH-coupled assay (Fig. 2c)^17^. The analysis indicated small positive cooperativity for the RecQ-ssDNA interaction, which was not systematically altered by the mutations (Hill coefficients were 1.7 ± 0.3, 1.0 ± 0.4 and 1.7 ± 0.3 for RecQ^WT^, RecQ^Y555A^ and RecQ^523^, respectively). In agreement with our previous results, *k*_cat_ was almost identical for RecQ^WT^ and RecQ^Y555A^, but was 3 fold-higher for RecQ^523^ (Table 1)^17^. The apparent dissociation constants for dT_54_ (*K*_d,app,dT54_), also determined from the dT_54_ concentration dependence of *k*_ss_ were slightly higher for RecQ^Y555A^ and RecQ^523^ compared to RecQ^WT^ (Table 1).

The apparent rate constant of ATP-induced DNA binding (*k*_b_, determined from lag kinetics using Supplementary equation (S1)) increased linearly with dT_54_ concentration (Supplementary Fig. S3). This dependence allowed another, independent means of determination of the ssDNA binding *K*_d_ values of RecQ constructs during ATPase cycling (Supplementary Fig. S3, Supplementary Table S1). The determined values (Supplementary Table S1) were slightly higher than those determined from *k*_ss_ values (Table 1), but were in line with those determined previously for the RecQ^WT^.AMPPNP (non-hydrolyzable ATP analog) complex^17^. The mutations caused a slight reduction in the ssDNA affinity of RecQ (Table 1, Supplementary Table S1). In summary, these results suggest that the ssDNA interaction of the HRDC domain has a minor contribution to the overall ssDNA binding affinity of RecQ, but the presence of the HRDC domain—regardless of its ssDNA binding capability—significantly suppresses the ssDNA-activated ATPase activity of the motor core.

To dissect how the HRDC domain affects ssDNA translocation, we monitored the kinetics of P_i_ generation from ATP hydrolysis during single-round translocation, which was previously shown to be suitable for the determination of translocation processivity and ATPase-translocation coupling (ATP hydrolyzed per nucleotide (nt) traveled)^24^,^25^,^26^,^29^.

**Figure 3:**
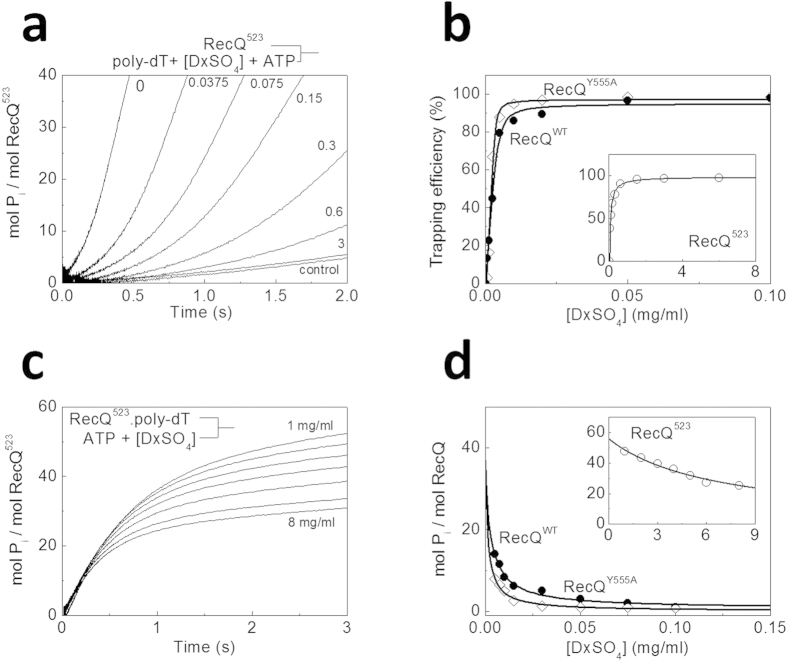
The HRDC domain does not influence the processivity of ssDNA translocation (**a**) Kinetics of P_i_ release observed upon mixing 20 nM RecQ^523^ with a pre-mixture of ATP (1 mM), poly-dT (25 μM, nt concentration) and various concentrations of DxSO_4_ (indicated in mg/ml) in the presence of 5 μM MDCC-PBP in the stopped-flow. The control trace was recorded in 6 mg/ml DxSO_4_ in the absence of poly-dT. The DxSO_4_ concentration dependence of steady-state ATP hydrolysis rates was used to determine the trapping efficiency (based on Supplementary equation (S2)) values shown in (**b**). Solid lines are hyperbolic fits to determine the DxSO_4_ concentrations required for half-maximal trapping (2.3 ± 0.1 μg/ml for RecQ^WT^, 1.8 ± 0.1 μg/ml for RecQ^Y555A^, and 62 ± 11 μg/ml for RecQ^523^). Symbols are as in Fig. 2c. (**c**) Time courses of P_i_ generation from ATP by RecQ^523^ during single-round translocation on poly-dT in the presence of 3 μM MDCC-PBP. 20 nM RecQ^523^ was pre-incubated with poly-dT (25 μM, nt) and mixed with ATP (1 mM) plus different concentrations of DxSO_4_ (1–8 mg/ml) in the stopped-flow apparatus. Traces consisted of an exponential and a linear phase, corresponding to ATP hydrolysis during translocation along poly-dT and in the DxSO_4_-bound end state, respectively. (**d**) DxSO_4_ concentration dependence of the amplitude of the translocation phase for all RecQ constructs. Symbols are as in Fig. 2c. Solid lines show fits based on Supplementary equation (S3). Determined parameters are listed in Table 1.

To ensure single-round translocation conditions, we used dextran sulfate (DxSO_4_) as a DNA-mimicking protein trap^25^. First we assessed the trapping efficiency of DxSO_4_ by rapidly mixing RecQ with a pre-mixture of ATP, poly-dT and different concentrations of DxSO_4_, and monitoring P_i_ generation using MDCC-PBP in a stopped-flow instrument. Poly-dT was used instead of oligo-dT to minimize DNA end effects. Similar to those recorded in the absence of DxSO_4_ (Fig. 2b), traces showed a lag followed by linear steady-state P_i_ generation (Fig. 3a). In the absence of DxSO_4_, the steady-state rate of P_i_ generation was 3 times higher for RecQ^523^ than for RecQ^WT^ and RecQ^Y555A^ (shown for RecQ^523^ in Fig. 3a), similar to the dT_54_ experiments of Fig. 2b–c (Table 1). As expected, the steady-state rate markedly decreased with increasing DxSO_4_ concentration (Fig. 3a). Surprisingly, we found that RecQ^523^ required 30-fold higher DxSO_4_ concentration for half-maximal inhibition of the ssDNA-activated steady-state ATPase activity than did RecQ^WT^ and RecQ^Y555A^ (Fig. 3b). This result suggested that the HRDC domain, regardless of its ssDNA-binding capability, is a crucial mediator of DxSO_4_ trapping of RecQ.

We characterized the ssDNA translocation processivity of RecQ constructs by determining the mean number of ATPase cycles during a single translocation run (<*n*_ATP_>, Table 1). In these experiments, RecQ was preincubated with poly-dT and then rapidly mixed with ATP and different concentrations of DxSO_4_ in a stopped-flow instrument. The kinetics of P_i_ generation during ATP hydrolysis was followed by MDCC-PBP. DxSO_4_ concentrations were chosen from the regime where the trapping efficiency was above 95% (cf. Fig. 3b). P_i_ generation kinetic traces comprised an exponential phase (characteristic of single-round ssDNA translocation) followed by a slow steady-state that ensued upon dissociation of RecQ from ssDNA after the translocation run (Fig. 3c). Thus, the amplitude of the exponential phase (mol P_i_/mol RecQ) equals <*n*_ATP_>. This amplitude decreased with increasing DxSO_4_ concentration for all RecQ constructs, indicating that DxSO_4_ actively facilitates RecQ dissociation from ssDNA during translocation. In the case of RecQ^523^, the amplitudes showed a shallower DxSO_4_ concentration dependence than for RecQ^WT^ and RecQ^Y555A^ (Fig. 3d), as expected based on the experiments of Fig. 3a–b. To determine the genuine (DxSO_4_-free) <*n*^0^_ATP_> value, we extrapolated to zero DxSO_4_ concentration using Supplementary equation (S3)^24^,^25^,^29^. RecQ^WT^ and RecQ^Y555A^ showed similar <*n*^0^_ATP_> values, whereas this value was slightly higher in RecQ^523^ (Table 1), reflecting that the HRDC domain does not have a profound effect on the ssDNA translocation processivity of RecQ.

**Figure 4:**
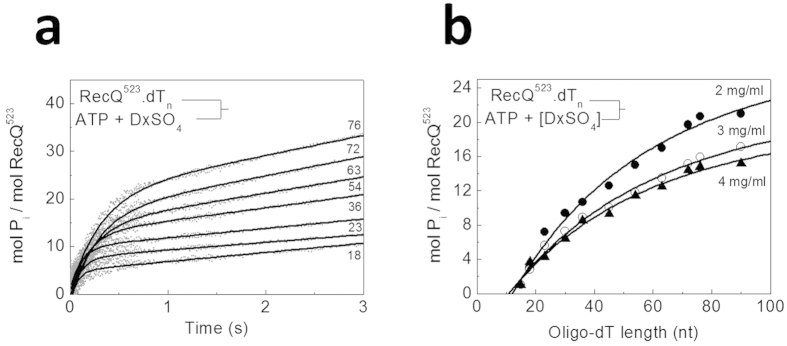
ATPase-translocation coupling in HRDC-deleted RecQ (**a**) Time courses of P_i_ generation from ATP during translocation of RecQ^523^ along oligo-dT substrates of different length (dT_15_ − dT_90_, lengths in nt are indicated), recorded upon mixing 20 nM RecQ^523^ plus dT_n_ (1 μM, except for dT_15_ (2.5 μM) and dT_18_ (2 μM)) with 1 mM ATP plus 2 mg/ml DxSO_4_ in the stopped-flow instrument. Traces comprised two phases as in Fig. 3c. (**b**), Oligo-dT length dependence of P_i_ generation amplitudes during the first (translocation-coupled) phase. Filled circles, empty circles and triangles represent values obtained in the presence of 2, 3 and 4 mg/ml DxSO_4_, respectively. Solid lines are fits based on Supplementary equation (S4). Determined parameters are listed in Table 1.

As described earlier, the ATPase-translocation coupling stoichiometry (*C*_trans_, number of ATP molecules hydrolyzed per nt traveled) can be determined from the ssDNA (oligo-dT) length dependence of P_i_ generation amplitudes during single-round ssDNA translocation^24^,^25^,^29^. In these experiments, we preincubated RecQ with oligo-dT substrates of different lengths and then rapidly mixed these premixtures with ATP plus DxSO_4_ in a stopped-flow instrument. Kinetic traces showed exponential and linear phases (Fig. 4a), similar to those observed for poly-dT (Fig. 3c). As expected, the single-round (exponential) P_i_ generation amplitudes increased with increasing oligo-dT length, showed saturation, and their maximal value decreased with increasing DxSO_4_ concentration (Fig. 4b). Fits to these data using Supplementary equation (S4) revealed a *C*_trans_ value of 1.0 ± 0.1 ATP/nt and an occluded site size (*b*) of 13 ± 1 nt for RecQ^523^ along ssDNA, both of which were independent of DxSO_4_ concentration in the assessed range (2
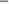
4 mg/ml) (Table 1). Comparison of these values with those determined earlier for RecQ^WT^ under identical or similar conditions revealed that deletion of the HRDC domain does not significantly affect the ATPase-translocation coupling but it decreases the occluded site size (Table 1)^25^,^26^.

The rate of ATP hydrolysis during translocation along poly-dT (*k*_ATP,trans_, determined from the initial slopes of the exponential phase in the experiments of Fig. 3c) was similar to those determined for DxSO_4_-free steady-state ATP hydrolysis for each construct (Fig. 3a, Table 1).

### The HRDC domain suppresses the rate of ssDNA translocation in parallel with that of ATP hydrolysis

The above results showed that the HRDC domain does not influence ATPase-translocation coupling (*C*_trans_), but it suppresses the rate of ATP hydrolysis during translocation (*k*_ATP,trans_). These findings imply that the rate of ssDNA translocation (*k*_trans_ (expressed in nt/s) = *k*_ATP,trans_/*C*_trans_) must also be suppressed by the HRDC domain, in parallel with the ATPase rate. Furthermore, we found that <*n*^0^_ATP_> was not greatly affected by the HRDC domain, implying that this domain neither affects the mean processive run length (nt traveled in a single run, <*n*_nt_> = <*n*^0^_ATP_>/*C*_trans_). As <*n*_nt_> = *k*_trans_/*k*_off,trans_ where *k*_off,trans_ is the rate constant of RecQ dissociation from ssDNA during translocation, one will expect that the latter parameter will be suppressed by the HRDC domain in parallel with the ATPase rate. Thus, the experimental determination of *k*_off,trans_ provides an independent means of verification of the proposed mechanochemical effects.

**Figure 5:**
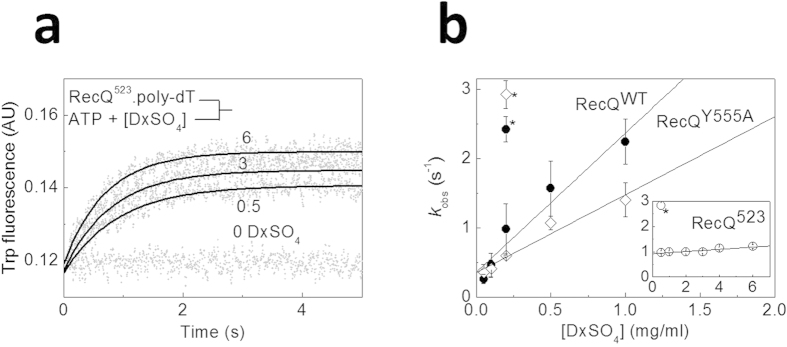
The HRDC domain suppresses the rate of dissociation from ssDNA during translocation (**a**) Trp fluorescence stopped-flow time courses monitoring the dissociation of RecQ^523^ (0.5 μM) from poly-dT (25 μM, nt) during single-round translocation initiated by rapidly mixing the enzyme-DNA complex with 1 mM ATP plus varying concentrations of DxSO_4_ (bottom to top: 0, 0.5, 3, and 6 mg/ml). Solid lines show single exponential fits. (**b**) DxSO_4_ concentration dependence of observed rate constants (*k*_obs_) determined for all three RecQ constructs as in (**a**). Solid lines show linear fits. The y intercept of the plots delineated the trap-free dissociation rate constant from poly-dT during translocation (*k*_off,trans_, Table 1). The slopes of the plots, characterizing the DxSO_4_-sensitivity of ssDNA dissociation, were 2.0 ± 0.3, 1.1 ± 0.1, and 0.04 ± 0.01 s^−1^(mg/ml)^−1^ for RecQ^WT^, RecQ^Y555A^, and RecQ^523^, respectively. Symbols marked by * indicate *k*_obs_ values determined in the absence of ATP. Symbols are as in Fig. 2c. AU, arbitrary units.

The intrinsic (tryptophan, Trp) fluorescence intensity of each ssDNA-bound RecQ construct is markedly lower than that of the DNA-free forms^17^. This signal is thus suitable for monitoring the kinetics of dissociation of RecQ molecules (i.e., *k*_off,trans_) from ssDNA upon completing single-round translocation^25^. We determined the *k*_off,trans_ values of RecQ constructs upon rapidly mixing the RecQ-poly-dT complex with excess ATP and varying concentrations of DxSO_4_ (Fig. 5a). The observed rate constants (*k*_obs_) of the transients increased linearly with DxSO_4_ concentration (Fig. 5b). The slopes and intercepts of the plots, reflecting the DxSO_4_ sensitivity of the reaction and the DxSO_4_-free *k*_off,trans_ value, respectively, were similar for RecQ^WT^ and RecQ^Y555A^ (Fig. 5b, Table 1). Importantly, the DxSO_4_-free *k*_off,trans_ of RecQ^523^ was 3 times higher than that of RecQ^WT^ and RecQ^Y555A^, providing independent verification of the above mechanochemical considerations.

As expected based on the experiments of Fig. 3, the ssDNA dissociation *k*_obs_ values of RecQ^523^ were less sensitive to DxSO_4_ compared with the other constructs (Fig. 5b, Table 1). In control experiments performed in the absence of ATP, *k*_obs_ values were several times higher than those in the presence of ATP at the corresponding DxSO_4_ concentration for all constructs (Fig. 5b). This finding indicates that the Trp fluorescence data obtained in the presence of ATP reliably report dissociation after single-round translocation. Calculated <*n*_nt_> values (=*k*_trans_/*k*_off,trans_) were slightly higher compared to those determined from MDCC-PBP experiments (<*n*_nt_> = <*n*^0^_ATP_>/*C*_trans_), but were practically identical for RecQ^WT^ and RecQ^523^ in both cases (Table 1).

### The HRDC domain does not affect the coupling between ATP hydrolysis and dsDNA unwinding

**Figure 6:**
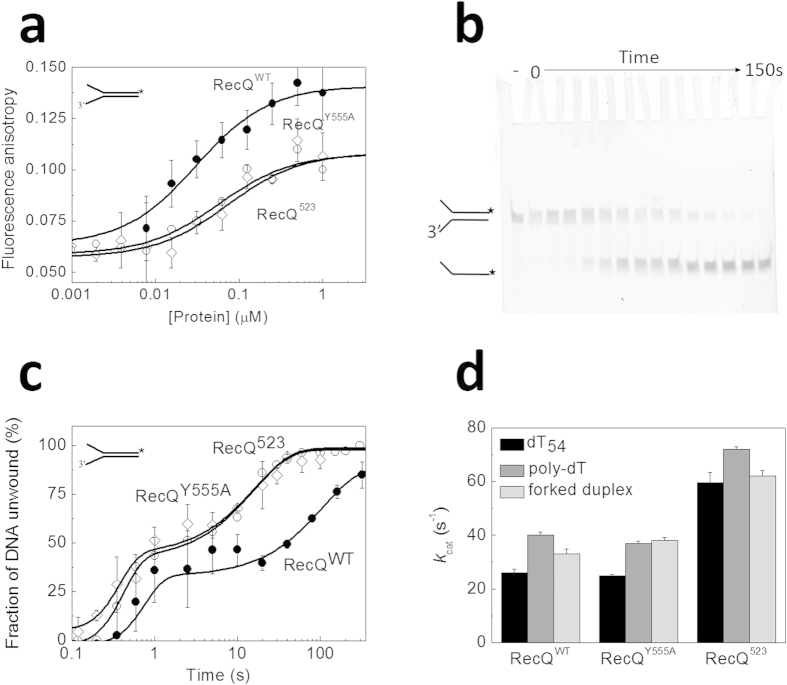
The HRDC domain modulates forked duplex DNA unwinding but does not affect ATPase-unwinding coupling (**a**) Fluorescence anisotropy titration of 10 nM fluorescein-labeled forked duplex DNA substrate (21-nt ssDNA arms + 33-bp dsDNA) with increasing amounts of RecQ constructs. Symbols are as in Fig. 2c. Solid lines show hyperbolic fits. Determined dissociation constants (*K*_d,forked duplex_) are listed in Table 1. (**b**) Electrophoretogram of a single-turnover unwinding experiment. Preincubation of fluorescein-labeled forked duplex (30 nM) with RecQ^523^ (100 nM) was followed by rapid mixing with ATP (3 mM) plus excess ssDNA trap strand (3 μM) (final post-mixing concentrations). Reactions were stopped by the addition of EDTA (40 mM) and loading dye at different time points (0–150 s; as in (**c**)) using a quench-flow instrument or by manual mixing. Amounts of DNA species (forked duplex and ssDNA, depicted by cartoons) labeled with fluorescein (asterisk) were detected by a fluorescence imager. “–“ denotes a 150-s control reaction in which ATP was absent. (**c**) Single-turnover unwinding kinetics of forked DNA (as in (**b**)). Symbols are as in Fig. 2c. Solid lines show fits based on Supplementary equation (S5). Determined parameters are listed in Table 1. (**d**) Steady-state ATPase *k*_cat_ (DNA-saturated ATPase) values of RecQ constructs (10 nM) in the presence of dT_54_, poly-dT and forked duplex DNA, determined in a PK-LDH coupled assay. Values obtained for dT_54_ and poly-dT are in line with those obtained in MDCC-PBP-based ATPase assays (Figs 2c and 3a; Table 1).

To determine the coupling between the ATPase and dsDNA unwinding activities, we devised experiments to measure the rate of DNA unwinding and the rate of ATPase activity in the presence of a forked duplex DNA substrate comprising 33 bp dsDNA with two 21-nt arms. First we determined the binding affinity of RecQ constructs to this DNA substrate by fluorescence anisotropy titrations utilizing a fluorescein label placed on the dsDNA-forming 3′-end of one strand (Fig. 6a). Compared to RecQ^WT^, the binding affinity (*K*_d, forked duplex_) was reduced about 4-5-fold in both RecQ^Y555A^ and RecQ^523^, reflecting a modest contribution of the HRDC-ssDNA interaction (Table 1).

To determine dsDNA unwinding rates, we performed rapid kinetic single-turnover unwinding experiments in which we rapidly mixed the RecQ-forked DNA complex with ATP and excess unlabeled ssDNA trap strand in a quenched-flow instrument, and monitored the time course of fluorescently-labeled ssDNA generation from forked duplex via gel electrophoresis of reaction products (Fig. 6b). Traces comprised a short (about 0.1-s) initial lag followed by two quasi-exponential rise phases in the case of all constructs (Fig. 6c), and were analyzed based on a previously described *n*-step kinetic model (Supplementary equation (S5))^32^. This model assumes that unwinding occurs as a result of *n* consecutive rate limiting steps that have a uniform rate constant. The model is suitable for the calculation of the macroscopic dsDNA unwinding rate (*k*_unw_) (Supplementary equation (S5)). The lag and the rapid unwinding phase of RecQ^WT^ was similar to that observed earlier using a Förster resonance energy transfer (FRET)-based assay^33^. We found that *k*_unw_ was slightly accelerated in RecQ^Y555A^ and RecQ^523^ compared to RecQ^WT^, indicating that the HRDC-ssDNA interaction moderately hinders dsDNA unwinding (Fig. 6c, Table 1).

The unwinding traces of all constructs contained an additional slow exponential phase (apparent after 5 s in Fig. 6c) (Table 1). As discussed earlier for RecBCD and UvrD helicases, this phase may result from a fraction of enzyme molecules bound to DNA non-productively, necessitating a rate-limiting initiation of unwinding^32^,^34^. Alternatively, if multiple helicase molecules are initially bound to the 3′-ssDNA overhang, the slow phase may result from the action of one helicase proceeding in the trail of the leading one^33^,^35^.

To assess ATPase-dsDNA unwinding coupling, we measured the steady-state ATPase rate of RecQ constructs (*k*_ATP,unw_) during unwinding of the same forked dsDNA substrate, using a pyruvate kinase-lactate dehydrogenase (PK-LDH) coupled assay (Fig. 6d). Compared to RecQ^WT^, the ATPase activity of RecQ^Y555A^ was slightly elevated, while RecQ^523^ had a significantly higher (*k*_ATP,unw_) value (Fig. 6d, Table 1). The calculated macroscopic ATPase-unwinding coupling stoichiometry (*C*_unw_ (expressed as ATP hydrolyzed per bp unwound) = *k*_ATP,unw_/*k*_unw_) was close to 1 ATP/bp for all constructs, suggesting that unwinding is tightly coupled to ATP hydrolysis and is not influenced by the HRDC domain (Table 1). Taken together, these results show similar trends to those found for ssDNA translocation: the HRDC domain hinders the rate of unwinding in parallel with that of the ATPase activity, without influencing the tight coupling between these processes.

## Discussion

Although the HRDC domain has generally been considered as an auxiliary ssDNA-binding element, recent crystal structures of human BLM constructs showed that the HRDC domain can fold back onto and interact with both RecA domains, both in the presence and absence of DNA (pdb ids.: 4CGZ, 4CGD, 4O3M) (Fig. 1c)^23^. These interdomain interactions were proposed to influence the ATPase activity and the coupled ssDNA translocation and dsDNA unwinding by BLM^23^. Indeed, suppression of DNA-activated ATPase activity by the HRDC domain was observed for various RecQ helicases (Table 2)^14^,^17^,^23^,^36^. Moreover, in the case of *Ec* RecQ, ATPase suppression is independent of the ssDNA-binding ability of the HRDC domain, further indicating interdomain interactions (RecQ^Y555A^ data in Table 1)^17^.

Importantly, however, the present study shows that the coupling of the RecQ ATPase activity to ssDNA translocation is unaffected by either the HRDC-ssDNA or the HRDC-motor core interactions. Moreover, the processivity of translocation is also unaffected by HRDC deletion in RecQ (Table 1), similar to the lack of effect of WHD-HRDC deletion on ATPase-translocation coupling and processivity in human BLM^14^.

We also found that the rate of forked duplex DNA unwinding, determined explicitly in transient kinetic experiments (Fig. 6c), is suppressed by the RecQ HRDC domain in parallel with the ATPase activity, indicating that coupling between these processes is not affected by the HRDC domain (Table 1). Interestingly, the DNA unwinding rate of RecQ^Y555A^ fell between those of RecQ^WT^ and RecQ^523^, indicating that HRDC-ssDNA interactions slightly suppress the rate of unwinding, unlike that of ssDNA translocation (Table 1).

Explicit kinetic rates of dsDNA unwinding have not been measured for HRDC-deletion constructs of other RecQ helicase homologs. Available steady-state unwinding kinetic^23^ and end-point measurements^14^,^36^ are not directly informative of possible changes in mechanochemical coupling, as they can be influenced by more complex features (unwinding processivity, unproductive initiation, reversal during unwinding etc.). Nevertheless, such data reflect the relative unwinding efficiencies of helicase constructs and indicate that the HRDC domain may affect ATPase-driven unwinding of forked duplex DNA by BLM in a way that is different from that in *Ec* and *Deinococcus radiodurans* (*Dr*) RecQ enzymes (Table 2)^22^,^36^. BLM constructs lacking the HRDC^23^ or both the WHD and HRDC domains^14^ showed decreased efficiency of forked duplex DNA unwinding despite increased ATPase activities (Table 2). Further studies are needed to clarify whether and how the varying properties of HRDC domains of different RecQ homologs contribute to mechanochemical coupling and/or more complex dynamic processes during dsDNA unwinding (see below).

RecQ helicases are thought to translocate along ssDNA and unwind dsDNA via ATP-driven inchworm-type stepping^24^,^25^,^26^. Thus, the interactions and the relative positions of the two RecA-like domains are likely to undergo coordinated changes during the ATP hydrolytic cycle, as reported for various other helicases harboring a similar motor core^37^,^38^. Based on the lack of direct polar interactions between the two RecA domains of BLM, it was proposed that the interactions of the HRDC domain with both RecA domains contribute to mechanochemical coupling^23^. HRDC deletion will therefore increase the flexibility of the RecA core, thereby accelerating the ATPase cycle but possibly decreasing ATPase-unwinding coupling efficiency^23^. However, other crystal structures (PDB codes 4CDG, 4CGZ) show polar interactions between the RecA domains of ADP-bound BLM, both in the DNA-free and the DNA-bound form. Interestingly, crystal structures of ADP- and DNA-bound ternary complexes of BLM show two different conformations (PDB codes 4CGZ and 4O3M)^23^. These two structures possibly represent different snapshots of the ATPase cycle, suggesting transient interactions between the two RecA domains and/or between the bound nucleotide and the C-core RecA domain. Similarly, the crystal structure of an *Ec* RecQ construct lacking the HRDC domain^22^ shows no direct polar interactions between the RecA domains in the absence of substrates, but such interactions are present in the ATPγS (pre-hydrolytic ATP analog) -bound form of the same construct.

Crystal structures of human RECQ1, a RecQ homolog naturally lacking the HRDC domain, also suggest inter-RecA communication via transient polar interactions^39^. RECQ1 bound to a dsDNA substrate with a 5-nt 3′-ssDNA overhang exhibits no direct polar inter-RecA interactions, while the C-core RecA domain interacts with DNA^39^. On the other hand, RECQ1 bound to dsDNA with a 4-nt 3′-ssDNA overhang contains an inter-RecA salt bridge, but shows no C-core RecA-DNA interaction (PDB code 4U7D). These findings reflect that inter-RecA coordination can be modified by interactions with the DNA substrate. Interestingly, the two RecA domains form numerous direct as well as water-mediated polar contacts in ADP-bound DNA-free RECQ1^39^. Together with the markedly (about 10 times) lower DNA-activated ATPase activity of RECQ1^40^ compared to *Ec* RecQ and human BLM^14^,^41^, this finding provides further indication for the inverse relationship between inter-RecA rigidity and ATPase kinetics. Thus, the HRDC-induced suppression of the ATPase activity of various HRDC-containing RecQ helicases (Table 2) is likely brought about by HRDC-mediated coordination of the RecA domains.

Our previous work showed that the similar macroscopic mechanochemical properties of *Ec* RecQ and human BLM result from different underlying kinetic mechanisms^24^,^30^. The steady-state rate of DNA-activated ATP hydrolysis by *Ec* RecQ is limited by the ATP cleavage step, whereas in BLM a transition between two ADP bound states is rate-limiting^24^,^30^. ATP hydrolysis by RecA-type ATPases is thought to be triggered by the so-called “arginine finger” residue that interacts with the γ-phosphate of the bound nucleotide in SF1 helicases^42^,^43^. The putative arginine finger located in the C-core RecA domain of different RecQ helicases shows variation in its nucleotide interactions, suggesting its possible role in kinetic tuning. The arginine finger does not interact with the bound nucleotide in DNA-free *Ec* RecQ·ATPγS^22^,^44^ and human RECQ1·ADP complexes^39^. In contrast, the arginine finger of DNA-bound BLM^45^ interacts with both phosphate groups of ADP (PDB code 4CGZ). These differences may indicate that the dynamic interaction of the arginine finger with the bound nucleotide during the hydrolytic cycle, which can in turn be influenced by HRDC-RecA core interactions, contributes to limiting the rate of enzymatic activity.

The HRDC domain of RecQ helicases is connected to the rest of the protein through a long and flexible loop, raising the possibility of dynamic interactions with the RecA core of the protein and/or the ssDNA regions of the DNA substrate. Amino acids involved in ssDNA binding by the isolated HRDC domain of BLM^18^ are buried in crystal structures of HRDC-containing BLM constructs^23^ due to the interaction with the RecA core, suggesting that the HRDC-ssDNA and HRDC-RecA core interactions are mutually exclusive.

Synthesis of our current findings on *Ec* RecQ with earlier data on BLM reveals that in both enzymes, depending on the structure of the DNA substrate encountered, the HRDC domain is prone to interact with ssDNA regions outside the DNA segment tracked by the RecA core. We found that the unwinding of forked DNA substrates by *Ec* RecQ is noticeably slowed by HRDC-ssDNA interactions (cf. RecQ^WT^ vs. RecQ^Y555A^ profiles in Fig. 6c, Table 1), but not by HRDC-RecA core interactions (cf. quasi-identical profiles of RecQ^Y555A^ and RecQ^523^ in the same experiment). Consistent with this observation, we found that the deletion of the HRDC domain drastically reduced the DxSO_4_ sensitivity of ssDNA translocation kinetics by *Ec* RecQ (Figs 3d and 5b), suggesting that the HRDC domain greatly assists ssDNA-RecQ-DxSO_4_ ternary complex formation. In parallel with these observations, the HRDC domain of BLM was suggested to bind to ssDNA regions outside the one tracked by the RecA core during unwinding of G4-containing DNA substrates^46^, and the HRDC domains of *Dr* RecQ and BLM were found to greatly contribute to recognition and processing of Holliday junction structures^36^,^47^.

On the other hand, in the presence of simple ssDNA substrates and absence of trap, the HRDC domain appears to dominantly interact with the RecA core, as inferred from the very small effects of the Y555A point mutation but marked effects of HRDC deletion under these conditions (Fig. 2c; and trap-free dissociation rate constants in Fig. 5b; Table 1). Taken together, available data suggest that the HRDC domain may dynamically switch between ssDNA- and RecA core-interacting modes, thereby fine-tuning DNA-restructuring processes.

## Materials and Methods

### Reagents

All reagents were from Sigma-Aldrich unless otherwise stated. ATP was from Roche Applied Science. Inorganic phosphate (P_i_) standard was from Merck. MDCC (7-diethylamino-3-((((2-maleimidyl)ethyl)amino)carbonyl)coumarin) was from Life Technologies. *ε*_260_ = 8400 M^−1^ cm^−1^ nt^−1^ was used for oligo-dT and poly-dT concentration determination. DNA concentrations are expressed as those of oligo- or polynucleotide molecules (as opposed to those of constituent nucleotide units (nt)) unless otherwise stated. Unless indicated otherwise, all experiments were performed at 25 °C in SF50 buffer (50 mM Tris-HCl pH 7.5, 50 mM NaCl, 1 mM DTT, 5 mM MgCl_2_, 50 μg/mL BSA).

### Protein expression and purification

RecQ variants were expressed and purified as described in ref. 17. Fluorescently labeled P_i_ binding protein (MDCC-PBP) was prepared as described in ref. 28.

### Circular dichroism (CD) spectroscopic measurements

UV (190–250 nm) CD spectra of proteins were recorded in a Jasco J-715 CD spectropolarimeter in a 1-mm path length cuvette. Measurements were done in SF50 buffer without BSA at 25 °C. Spectra were recorded three times independently and were averaged. Data were corrected with the CD spectra of BSA-free SF50 buffer.

### dsDNA substrates

Forked duplex DNA substrates were generated as follows. Equimolar amounts of oligonucleotides fork1 (5′-CGTTGCGCAATTAAGCTCTAAGCCATCCGCAAATTTTTTTTTTTTTT-TTTTTTT-3′) and fork2 (5′-TCCTTTTGATAAGAGGTCATTTTTGCGGATGGTTAGAGCTTAATTG-CGCAACG-Fluorescein) were mixed in a buffer comprising 10 mM Tris-HCl (pH 7.5) and 50 mM NaCl. Samples were heated to 100 °C and were left to cool down to room temperature overnight. The annealed DNA mixtures were purified on a MonoQ anion exchange column using a 0.01–1 M NaCl gradient for elution. Eluted fractions were analyzed by PAGE. Fractions containing the desired DNA structures were desalted by using an Amicon Ultra centrifuge filter (Millipore). DNA substrates were aliquotted and stored at –80 °C. The ssDNA trap strand used in single-round unwinding experiments is the unlabeled version of the fork2 oligonucleotide.

### PK-LDH-coupled ATPase measurements

Steady-state ATPase experiments were carried out in SF50 buffer by using a pyruvate kinase-lactate dehydrogenase (PK-LDH) coupled assay (14 U/mL PK, 20 U/mL LDH, 1 mM ATP, 1 mM phosphoenol pyruvate, 200 μM NADH). Time courses of NADH absorbance (ε_340_ = 6220 M^−1^cm^−1^) were followed in a Shimadzu UV-2101PC spectrophotometer.

### Stopped-flow measurements

Stopped-flow measurements were carried out in a BioLogic SFM 300 instrument. Post-mixing concentrations are stated. Trp fluorescence was detected through a 320-nm cutoff filter at 280-nm excitation with a 4-nm bandwidth. P_i_ generation from ATP was followed using MDCC-PBP. The setup for MDCC-PBP calibration and single-round translocation experiments were as described earlier^24^,^25^,^30^.

### Fluorescence anisotropy titrations

10 nM of forked duplex substrate was titrated with increasing concentrations of RecQ constructs. Fluorescence anisotropy was measured in a Synergy H4 Hybrid Multi-Mode Microplate Reader (BioTek).

### DNA unwinding experiments

In single-turnover unwinding experiments DNA substrates (30 nM; final reaction concentrations stated) were incubated with excess enzyme (100 nM) at 25 °C for 5 minutes in Buffer H (30 mM Tris-HCl pH 7.5, 100 mM KCl, 1 mM DTT, 50 μg/ml BSA, 20 mM creatine phosphate, 20 μg/ml creatine kinase). Reactions were started at 25 °C by mixing the DNA-enzyme complex with Buffer H containing ATP (3 mM), MgCl_2_ (3 mM) and ssDNA trap strand (3 μM) to provide single-turnover conditions. Reactions were stopped after pre-set reaction times by the addition of EDTA (40 mM final, pH 8.1) and loading dye (10 mM Tris-HCl pH 7.5, 40 mM EDTA, 60% v/v glycerol, 0.075% w/v Orange G, 0.83% w/v SDS). Reaction times shorter than 5 seconds were achieved by using a rapid quench-flow instrument (RQF-3, KinTek). Longer reactions were started and stopped manually. Post-reaction mixtures were incubated at 25 °C for additional 3 min and were held on ice until further processing. Samples were then loaded on 12% non-denaturing polyacrylamide gels in TBE buffer (89 mM Tris-HCl pH 7.5, 89 mM boric acid, 20 mM EDTA). Electrophoresis was carried out at 4 °C. Fluorescently-labeled DNA was detected by using a Typhoon TRIO+Variable Mode Imager (Amersham Biosciences). The intensities of bands corresponding to the DNA substrate and unwinding products were quantified by densitometry (GelQuant Pro software (DNR Bio Imaging Ltd.)).

### Data analysis

Means ± SEM values (*n* = 3) are reported in the paper, unless otherwise specified. Data analysis was performed using OriginLab 8.0 (Microcal corp.).

## Additional Information

**How to cite this article**: Harami, G.M. *et al.* The HRDC domain of *E. coli* RecQ helicase controls single-stranded DNA translocation and double-stranded DNA unwinding rates without affecting mechanoenzymatic coupling. *Sci. Rep.* **5**, 11091; doi: 10.1038/srep11091 (2015).

## Supplementary Material

Supplementary Information

## Figures and Tables

**Table 1:** **Mechanistic parameters of RecQ constructs.**

	**RecQ^WT^**	**RecQ^Y555A^**	**RecQ^523^**
ATPase activity and DNA binding			
*k*_cat,dT54_ (s^−1^)^a^,^b^	25 ± 3 (28 ± 2)	26 ± 2 (27 ± 1)	86 ± 2 (59 ± 1)
*k*_cat,poly-dT_ (s^−1^)^a^,^b^	35 ± 2 (40 ± 1)	32 ± 1 (37 ± 1)	88 ± 5 (72 ± 1)
*k*_basal_ (DNA-free, s^−1^)^a^	0.32 ± 0.01	0.30 ± 0.01	0.38 ± 0.01
*k*_cat,DxSO4_ (s^−1^)^a^	0.57 ± 0.01	0.64 ± 0.01	0.95 ± 0.02
*K*_d,app,dT54_ (μM, nt)^a^	1.2 ± 0.2	3.1 ± 0.3	2.2 ± 0.3
*K*_d,forked duplex_ (nM)^c^	13 ± 4	46 ± 2	60 ± 2
ssDNA translocation			
*C*_trans_ (coupling stoichiometry, ATP hydrolyzed per nt traveled)^d^	0.6 ± 0.2^e^; 1.1 ± 0.2^f^	n.d.	1.0 ± 0.1
*k*_ATP,trans_ (rate constant of ATP hydrolysis during translocation, s^−1^)^g^	29 ± 1 (~10^e^; 35±4^f^)	30 ± 6	70 ± 2
*k*_trans_ (translocation rate, nt/s)^h^	26 ± 5 (16±4^e^; 31±8^f^)	n.d.	70 ± 7
*k*_off,trans_ (DNA dissociation rate constant during translocation, s^−1^)^i^	0.35 ± 0.14 (0.43±0.04^e^; 0.12±0.01^f^)	0.34 ± 0.13	0.92 ± 0.05
<*n*^0^_ATP_>(mean number of ATPase cycles in a single run)^j^	41 ± 5	37 ± 7	56 ± 1
<*n*_nt_>(mean number of nt traveled in a single run)	37 ± 8^k^; 74 ± 32^l^ (36±2^e^; ~100^f^)	n.d.	56 ± 6^k^; 76 ± 8^l^
*P* (processivity)^m^	0.973; 0.986 (0.972^e^; 0.991^f^)	n.d.	0.982; 0.987
Occluded site size (nt)^d^	34 ± 3e; 18 ± 2f	n.d.	13 ± 1^g^
dsDNA unwinding			
*k*_kin_ (rate constant of kinetic step, s^−1^)^n^	5.3 ± 1.1	8.2 ± 1.5	10.1 ± 2.2
*k*_unw_ (macroscopic unwinding rate, bp/s)^n^	33 ± 7	51 ± 9	63 ± 12
*k*_2_ (rate constant of slow phase, s^−1^)^n^	0.0090 ± 0.0020	0.059 ± 0.012	0.064 ± 0.015
*k*_ATP,unw_ (rate constant of ATP hydrolysis during unwinding, s^−1^)^o^	33 ± 2	38 ± 1	62 ± 2.2
*C*_unw_ (coupling stoichiometry, ATP hydrolyzed per bp unwound)^p^	1.0 ± 0.2	0.75 ± 0.12	1.0 ± 0.2

N.d., Not determined.

^a^From P_i_ release data.

^b^Values in parentheses were determined from PK-LDH-coupled assays.

^c^From fluorescence anisotropy titrations.

^d^From oligo-dT length dependence of P_i_ release amplitudes during single-round translocation.

^e^From ref. 26.

^f^From ref. 25.

^g^From the initial slope of poly-dT translocation experiments.

^h^Calculated as *k*_ATP,trans_/*C_trans_*.

^i^From Trp fluorescence data.

^j^From DxSO_4_ dependence of P_i_ release amplitudes during single-round translocation on poly-dT.

^k^Calculated as <*n*^0^_ATP_>/*C_trans_*.

^l^Calculated as *k*_trans_/*k*_off,trans_.

^m^Calculated as <*n*_nt_>/(<*n*_nt_> + 1).

^n^From single-turnover unwinding experiments.

^o^From PK-LDH-coupled assays.

^p^Calculated as *k*_ATP,unwinding_*/k*_unw_.

**Table 2:** **Comparison of effects of HRDC domain mutation/deletion in RecQ helicases.**

	**ATPase activity (relative to WT)**	**Unwinding efficiency (relative to WT)**
***Ec*****RecQ** (RecQ^WT^)	1	1
Y555A (RecQ^Y555A^)	1.2	1.5^a^
dHRDC (RecQ^523^)	1.9	1.9^a^
**BLM** (BLM^642–1290^^b^, BLM^636–1298^^c^)^d^	1	1
dHRDC (BLM^636–1195^)^c^	3.7	0.11^e^
dWHD-dHRDC (BLM^642–1077^)^b^	1.5	0.19^f^
***Dr*** **RecQ** (WT containing 3 tandem HRDC domains (HRDC_1–3_), DrRecQ^1–824^)^g^	1	1
dHRDC_3_ (Dr RecQ^1–728^)	1.8^h^	1^f^,^i^
dHRDC_2–3_ (Dr RecQ^1–610^)	2.1^h^	~5^f^,^i^
dHRDC_1–3_ (Dr RecQ^1–519^)	>2.5^h^	~5^f^,^i^

Data were obtained using forked duplex DNA if not otherwise indicated. *Ec* RecQ data are from this study. “dWHD” and “dHRDC” refer to winged-helix domain and HRDC domain deletion, respectively.

^a^Based on unwindin*k*g rates determined in single-turnover experiments.

^b^From ref. 14.

^c^From ref. 23.

^d^Monomeric constructs considered as WT, comprising all domains present in *Ec* RecQ.

^e^Based on steady-state unwinding rates.

^f^Based on enzyme concentration required for half-maximal unwinding efficiency.

^g^From ref. 36.

^h^dT_28_ data.

^i^3′-tailed dsDNA substrate.
