# Supplementary material for: The HRDC domain of E. coli RecQ helicase controls single-stranded DNA translocation and double-stranded DNA unwinding rates without affecting mechanoenzymatic coupling
Source: Sci Rep. 2015 Jun 11;5:11091. doi: 10.1038/srep11091 (PMC4464074; doi:10.1038/srep11091)
Supplement: Supplementary Information [file srep11091-s1.pdf]

# The HRDC domain of *E.coli* RecQ helicase controls single-stranded DNA translocation and double-stranded DNA unwinding rates without affecting mechanoenzymatic coupling

Gábor M. Harami[1], Nikolett T. Nagy[1], Máté Martina[1], Keir C. Neuman[2], Mihály Kovács[1,]*

[1] Department of Biochemistry, ELTE-MTA "Momentum" Motor Enzymology Research Group, Eötvös Loránd University, Pázmány P. s. 1/c, H-1117 Budapest, Hungary

[2] Laboratory of Molecular Biophysics, National Heart, Lung and Blood Institute, National Institutes of Health, Bethesda, Maryland 20892, USA

* Corresponding author. Email: mihaly.kovacs@ttk.elte.hu.

## Supplementary Tables

### Supplementary Table S1. DNA binding parameters of RecQ constructs

| | **RecQ$^{WT}$** | **RecQ$^{Y555A}$** | **RecQ$^{523}$** |
|---|---|---|---|
| $k_{on}$ ((µM, nt)$^{-1}$s$^{-1}$) [a] | 0.12 ± 0.01 | 0.09 ± 0.01 | 0.13 ± 0.01 |
| $k_{off}$ (s$^{-1}$) [a] | 0.55 ± 0.27 | 0.51 ± 0.21 | 0.70 ± 0.43 |
| $K_d$ (µM, nt) [b] | 4.6 ± 2.3<br>(3.0 ± 1.7 [c] ; 7.0 ± 0.8 [d]) | 5.7 ± 2.4 | 5.4 ± 3.3 |

All data were obtained using $dT_{54}$.

[a] Determined from data presented in **Supplementary Fig. S3**.

[b] Calculated as $k_{off}/k_{on}$.

[c] Determined in the presence of AMPPNP[1].

[d] Determined for nucleotide-free RecQ[1].

## Supplementary Figures

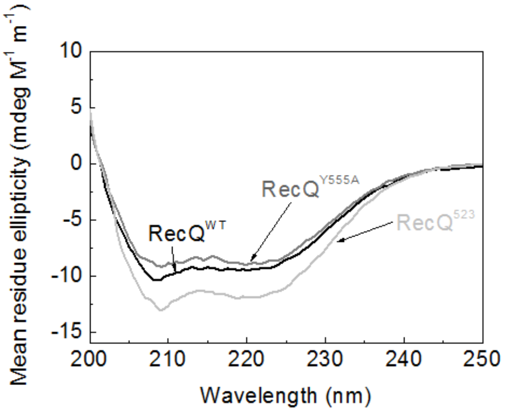


**Supplementary Figure S1. CD spectra indicate proper folding of RecQ constructs**

CD spectra of $RecQ^{WT}$ (black), $RecQ^{Y555A}$ (dark gray) and $RecQ^{523}$ (gray) are characteristic of folded proteins with a mixed α/β structure.

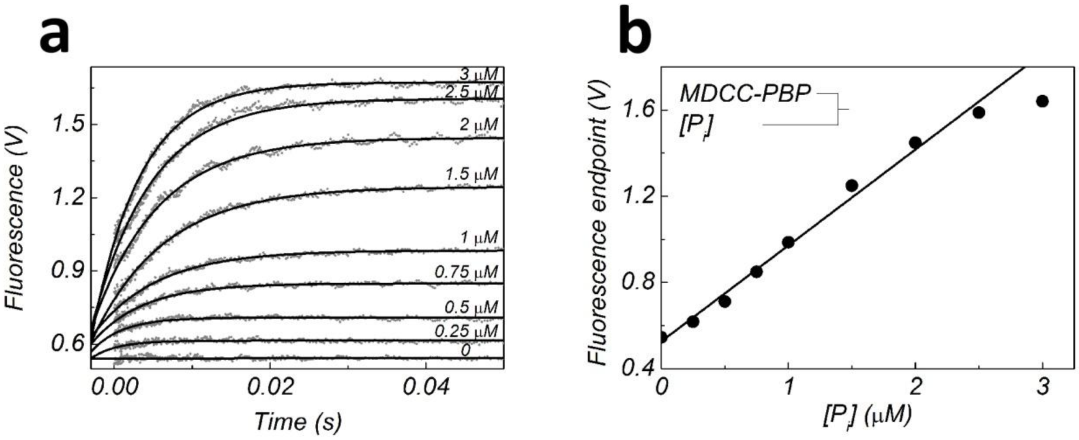


**Supplementary Figure S2. MDCC-PBP fluorescence calibration**

(**a**) Kinetic traces of fluorescence emission change of MDCC-PBP (3 μM) upon rapidly mixing with the indicated $P_i$ concentrations in the stopped-flow. Solid lines show single exponential fits. (**b**) $P_i$ concentration dependence of maximal MDCC-PBP fluorescence emission. Fluorescence endpoint values showed a plateau due to saturation of MDCC-PBP with $P_i$. Solid line shows a linear fit to the first 8 data points. The slope of the fitted line was used for conversion of MDCC-PBP fluorescence to $P_i$ concentration.

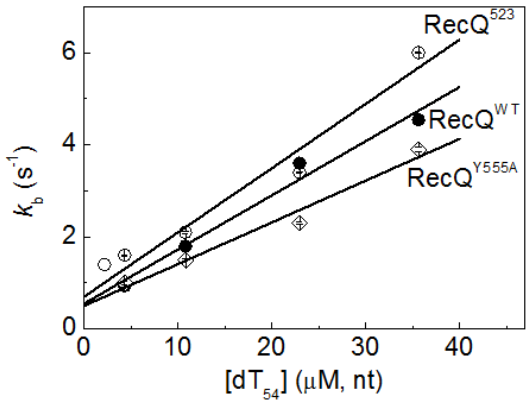


**Supplementary Figure S3. DNA binding kinetics of RecQ constructs determined from ATPase measurements**

Shown is the $dT_{54}$ concentration dependence of apparent $dT_{54}$ binding rate constants ($k_b$ in **Supplementary equation (S1)**) of RecQ constructs, determined from experiments of **Fig. 2b**. Symbols are as in **Fig. 2c**. Solid lines show linear fits ($k_b = k_{on}[DNA] + k_{off}$) with slopes yielding apparent second-order ssDNA binding rate constants ($k_{on}$) and intercepts reflecting ssDNA dissociation rate constants ($k_{off}$) listed in **Supplementary Table S1**. Values for $RecQ^{WT}$ were in line with those determined for a nucleotide-dependent isomerization in Trp fluorescence experiments[1].

## Supplementary Equations

### Supplementary Equation S1. DNA concentration dependence of pre-steady-state and steady-state $P_i$ generation resulting from ATP hydrolysis

Kinetic traces of $P_i$ generation from ATP by RecQ constructs (**Fig. 2b**) consisted of a lag followed by linear steady-state $P_i$ generation. Based on earlier results[1], the lag likely originates from a pre-steady-state increase in the fraction of DNA-bound RecQ molecules, which is triggered by ATP binding. Previous results on ATP binding kinetics[2] showed that ATP binding to RecQ occurs rapidly (>300 $s^{-1}$) at the applied ATP concentration (1 mM) and thus does not influence the kinetic traces of **Fig. 2b**. Therefore, at any given time point ($t$), the ratio ($P_i$ release rate)/[total RecQ] will equal the product of the catalytic rate constant ($k_{cat}$) and the actual fraction of DNA-bound RecQ molecules ([DNA-bound RecQ]($t$)/[total RecQ]). The time course of the latter fraction was approximated to follow reversible pseudo-first-order binding kinetics: [DNA-bound RecQ]($t$)/[total RecQ] = $\alpha(1-\exp(-k_b t))$ where $k_b$ is the apparent rate constant of DNA binding, and α is the steady-state fraction of DNA-bound RecQ molecules at the given DNA concentration. Thus, the time course of $P_i$ generation will follow

$$\frac{mol\ P_i}{mol\ RecQ}(t) = \frac{k_{ss}}{k_b} \cdot e^{-k_b \cdot t} + k_{ss} \cdot t - \frac{k_{ss}}{k_b} \qquad \text{(S1)}$$

where $k_{ss}$ (= $\alpha k_{cat}$) is the steady-state ATPase activity at the given DNA concentration. The DNA concentration dependence of determined $k_{ss}$ values is shown in **Fig. 2c**. **Table 1** shows the determined $k_{cat}$ and $K_{d,app,dT54}$ (apparent $dT_{54}$ dissociation constant during ATP hydrolysis) values.

$k_b$, in turn, will depend on DNA concentration as $k_b = k_{on}[DNA] + k_{off}$ where $k_{on}$ and $k_{off}$ are the on- and off-rate constants of DNA binding, respectively. **Supplementary Fig. S3** shows the DNA concentration dependence of $k_b$ in the pseudo-first order DNA concentration regime, and **Supplementary Table S2** shows the determined $k_{on}$ and $k_{off}$ values.

### Supplementary Equation S2: Calculation of the trapping efficiency of $DxSO_4$

Steady-state poly-dT-activated ATPase activities at different $DxSO_4$ concentrations (**Fig. 3a**) were used to calculate the trapping efficiencies at each $DxSO_4$ concentration as

$$\text{Trapping efficiency}(\%) = 100 \cdot \frac{ATPase\ activity - k_{\text{cat,DxSO}_4}}{k_{\text{cat,poly-dT}} - k_{\text{cat,DxSO}_4}} \qquad \text{(S2)}$$

where $k_{cat,poly-dT}$ is the $DxSO_4$-free poly-dT activated ATPase activity, and $k_{cat, DxSO4}$ is the ATPase activity of $DxSO_4$-saturated RecQ. Hyperbolic fits to the $DxSO_4$ concentration dependence of the trapping efficiency (**Fig. 3b**) were used to determine the $DxSO_4$ concentration required for half-maximal trapping efficiency (listed in the legend to **Fig. 3b**).

**Supplementary Equation S3: Equation used for determining the trap ($DxSO_4$) concentration dependence of translocation processivity along poly-dT**

For the analysis of the data in **Fig. 3d**, we used the equation described previously in ref. [3], in which the mean number of ATPase cycles performed during single-round translocation at a given trap concentration ($\langle n_{ATP} \rangle$ ([*T*])) is given as

$$\langle n_{ATP} \rangle ([T]) = \frac{K_d^{'} + [T]}{\dfrac{K_d^{'}}{\langle n_{ATP}^0 \rangle} + \dfrac{[T]}{\langle n_{ATP}^T \rangle}} \qquad \text{(S3)}$$

where $\langle n^0_{ATP} \rangle$ and $\langle n^T_{ATP} \rangle$ are the mean number of ATPase cycles performed in the presence of zero and saturating trap concentrations, respectively; [*T*] is the concentration of the $DxSO_4$ trap; and $K'_d$ is the apparent dissociation constant for $DxSO_4$ binding to the enzyme. The trap-free processivity (*P*), defined as the probability of performing the next translocation step (as opposed to dissociation from the ssDNA track) is calculated as $\langle n^0_{ATP} \rangle / (\langle n^0_{ATP} \rangle + 1)$ (**Table 1**).

**Supplementary Equation S4: Equation used for analysis of the oligo-dT length dependence of $P_i$ generation amplitudes during single-round unwinding**

For the analysis of the data in **Fig. 4b**, we used the equation described previously in refs. [3-5] :

$$A_{rand}(L) = \langle n_{ATP} \rangle \cdot \left( 1 - \frac{1}{C \cdot (L-b) + 1} \cdot \frac{1 - \left( \dfrac{\langle n_{ATP} \rangle}{\langle n_{ATP} \rangle + 1} \right)^{C \cdot (L-b)+1}}{1 - \dfrac{\langle n_{ATP} \rangle}{\langle n_{ATP} \rangle + 1}} \right) \qquad \text{(S4)}$$

where $A_{rand}$ (*L*) is the amplitude of $P_i$ release (mol $P_i$/mol RecQ, equal to ATP consumption) during single-round translocation starting from a random position on an oligo-dT substrate of length *L* (nt), $\langle n_{ATP} \rangle$ is the mean number of ATPase cycles during single round translocation, *C* is the macroscopic coupling stoichiometry (ATP molecules hydrolyzed/nt traveled), and *b* is the occluded site size of the

helicase on the ssDNA track. Determined <$n_{atp}$> values were 31 ± 1, 25 ± 1, and 22 ± 1 at 2, 3, and 4 mg/ml $DxSO_4$, respectively.

**Supplementary Equation S5: Equation used for determining the dsDNA unwinding rate from single-turnover experiments**

For the analysis of the data in **Fig. 6c**, we used the equation described previously in ref. [6]. The model assumes that the appearance of the fully unwound DNA species (i.e. ssDNA in the experiment) results from *n* consecutive rate-limiting steps (so-called kinetic steps) with a uniform rate constant $k_{kin}$. Thus, the fraction of DNA unwound at a given time point ($U(t)$) can be calculates as

$$U(t) = A\left(1 - \sum_{i=1}^{n} \frac{{k_{\mathrm{kin}}}^{i-1} \cdot t^{i-1}}{(i-1)!} \cdot e^{-k_{\mathrm{kin}} \cdot t}\right) + B\left(1 - e^{-k_2 \cdot t}\right) \qquad \text{(S5)}$$

where *A* and *B* are the amplitudes of the rapid and slow unwinding phases, respectively (expressed in fraction of DNA molecules), and $k_2$ is the observed rate constant of the slow phase. We performed fits using an integer series of *n* ranging from 1 to 10, while all other parameters were allowed to float. For all constructs, the smallest $\chi^2$ value was obtained at $n = 4$. The apparent kinetic step size, i.e. the number of base pairs unwound in a kinetic step can be calculated as $L/n$ where *L* is the effective length of the enzymatically unwound dsDNA stretch (in bp). The forked duplex substrate used had a dsDNA segment of 33 bp. However, *L* will be shorter than this value, due to spontaneously unwinding of the dsDNA end. Based on ref. [7] using melting temperature calculations, the last 8 bp of the substrate will unwind spontaneously at 25°C with around 50 % probability. Accordingly, we considered *L* to be 25 bp, yielding an apparent kinetic step size around 6 bp for all constructs. The macroscopic unwinding rate ($k_{unw}$, expressed in bp/s; **Table 1**) was estimated as the product of $k_{kin}$ and the apparent unwinding step size.

We note that $k_{unw}$ represents an upper bound for the unwinding rate, as we omitted the rate of dissociation from DNA in the calculations. (The introduced error is probably small as the rate constant of dissociation during processive unwinding is expected to be much smaller than that of the unwinding step.) The precise microscopic mechanism of unwinding could be determined by more extensive experimentation[8,9]. However, the applied analysis is fully suitable for the detection of any significant mutation-induced change in the unwinding activity (see Results).

## Supplementary References

1. Kocsis, Z. S., Sarlós, K., Harami, G. M., Martina, M. & Kovács, M. A Nucleotide-dependent and HRDC Domain-dependent Structural Transition in DNA-bound RecQ Helicase. *J Biol Chem.* **289**, 5938-5949 (2014).

2. Sarlós, K., Gyimesi, M., Kele, Z. & Kovács, M. Mechanism of RecQ helicase mechanoenzymatic coupling reveals that the DNA interactions of the ADP-bound enzyme control translocation run terminations. *Nucleic Acids Res.* **43**, 1090-1097 (2015).

3. Sarlós, K., Gyimesi, M. & Kovács, M. RecQ helicase translocates along single-stranded DNA with a moderate processivity and tight mechanochemical coupling. *Proc Natl Acad Sci U S A.* **109**, 9804-9809 (2012).

4. Gyimesi, M., Sarlós, K. & Kovács, M. Processive translocation mechanism of the human Bloom's syndrome helicase along single-stranded DNA. *Nucleic Acids Res.* **38**, 4404-4414 (2010).

5. Gyimesi, M., Sarlós, K., Derényi, I. & Kovács, M. Streamlined determination of processive run length and mechanochemical coupling of nucleic acid motor activities. *Nucleic Acids Res.* **38**, e102 (2010).

6. Ali, J. A. & Lohman, T. M. Kinetic measurement of the step size of DNA unwinding by Escherichia coli UvrD helicase. *Science.* **275**, 377-380 (1997).

7. Donmez, I., Rajagopal, V., Jeong, Y. J. & Patel, S. S. Nucleic acid unwinding by hepatitis C virus and bacteriophage t7 helicases is sensitive to base pair stability. *J Biol Chem.* **282**, 21116-21123 (2007).

8. Lucius, A. L., Maluf, N. K., Fischer, C. J. & Lohman, T. M. General methods for analysis of sequential "n-step" kinetic mechanisms: application to single turnover kinetics of helicase-catalyzed DNA unwinding. *Biophys J.* **85**, 2224-2239 (2003).

9. Fischer, C. J., Tomko, E. J., Wu, C. G. & Lohman, T. M. Fluorescence methods to study DNA translocation and unwinding kinetics by nucleic acid motors. *Methods Mol Biol.* **875**, 85-104 (2012).
